# A DFT Study of the Photochemical Dimerization of Methyl 3-(2-Furyl)acrylate and Allyl Urocanate

**DOI:** 10.3390/molecules191220482

**Published:** 2014-12-08

**Authors:** Maurizio D’Auria

**Affiliations:** Dipartimento di Scienze, Università della Basilicata, Viale dell’Ateneo Lucano 10, 85100 Potenza, Italy; E-Mail: maurizio.dauria@unibas.it; Tel.: +39-0971-205480; Fax: +39-0971-205678

**Keywords:** photochemical dimerization, 3-(2-furyl)acrylates, urocanates, cinnamates, DFT

## Abstract

A DFT study of the photochemical dimerization of methyl 3-(2-furyl)acrylate is reported. The photochemical reaction gave a mixture of two dimers with high regioselectivity and good stereoselectivity. Calculations showed that benzophenone was able to act as a photosensitizer of the reaction. This compound populated the first excited triplet state of the substrate. The frontier orbitals interaction between LSOMO of the triplet state and HOMO of the ground state accounted for the observed high regioselectivity. Furthermore, the energy of all the possible triplet biradicals has been calculated, showing that the precursor of the main product was the triplet biradical with the lowest energy. The coupling of the atomic coefficients on the radical centres in the biradical intermediates allowed to justify the observed products. The same behavior was observed in the case of the photochemical dimerization of an urocanate ester and in the dimerization of liquid methyl cinnamate.

## 1. Introduction

The dimerization of conjugated double bonds is one of most ancient known photochemical reactions, as Ciamician published his results on the solid phase photodimerization of cinnamic acid, stilbene, and coumarin in 1902 [1,2].

**Scheme 1:**
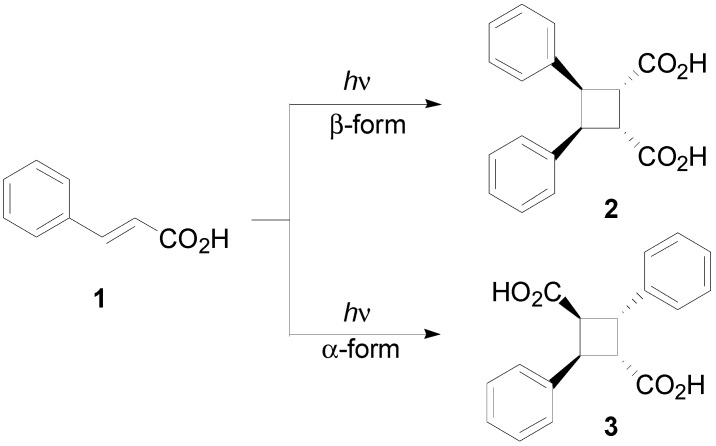
Photodimerization of cinnamic acid.

The dimerization of cinnamic acid derivatives has been recently reviewed [3]. Cinnamic acid (**1**), irradiated in the solid state, gave the corresponding dimers depending on the crystal form of the starting material: the metastable β-form was reported to yield β-truxinic acid (**2**), while the stable α-form gave α-truxillic acid (**3**) (Scheme 1) [4,5,6,7,8,9,10]. Recently, solid-state NMR analysis of this reaction has been performed [11].

**Scheme 2:**
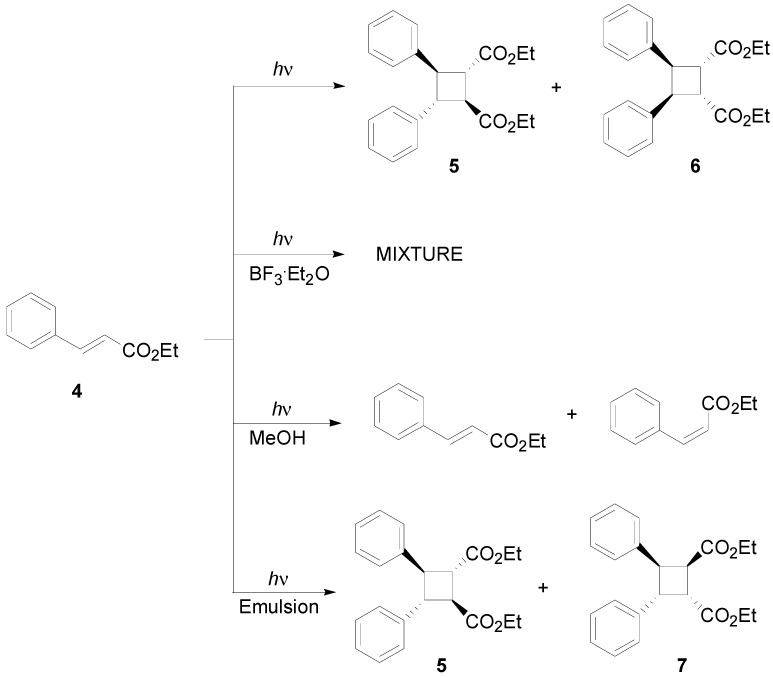
Photodimerization of ethyl cinnamate.

The same type of photodimers was obtained when the reaction was performed on 3-(2-furyl)acrylic acid or on 3-(2-thienyl)acrylic acid in the solid state [12]. The irradiation of the same compounds in methanol showed only the *E-Z* isomerization of the starting materials [13]. The irradiation of liquid ethyl cinnamate (**4**) furnished a mixture of two compounds, **5** and **6**, in 55% and 25% yields, respectively (Scheme 2) [14,15].

When the reaction was performed in a mixture of water (82.1%), cyclohexane (3.2%), butanol (9.8%), and sodium dodecyl sulfate (4.9%), an 8:2 mixture of the *trans*-diesters **5** and **7** was obtained (Scheme 2) [16]. On the other hand, the irradiation in methanolic solution did not furnish any dimerization product, giving instead only *E-Z* isomerization [17], while, the irradiation in the presence of BF_3_, furnished a mixture of seven dimers (Scheme 2) [18,19]. However, the application of this methodology to methyl 3,4-dimethoxycinnamate failed [20]. Cyclodextrin was used in order to perform the photochemical dimerization of 3,4-dimethoxycinnamic derivatives [21]. However, the irradiation of methoxy-, dimethoxy-, and trimethoxycinnamate esters gave the corresponding dimers in acetonitrile both in the presence or in absence of a triplet sensitizer [22]. Recently, dimerization in solution of cinnamic acid derivatives has been attempted by using γ-cyclodexrin [23], cucurbiturils [23,24], or Pd nanocage [25]. Furthermore, the role of ammonium ion to induce a supramolecular assembly of cinnamic acid has been studied [26].

Dimers of cinnamic acid and its derivatives were found in several plants and showed some interesting biological properties [27,28,29,30].

Some years ago we reported that the irradiation of a solution of furylacrylate esters in the presence of benzophenone gave the corresponding dimers with high regioselectivity and good stereoselectivity [31,32].

Some years later, we showed that allyl urocanate under the same conditions gave the corresponding dimer with high regio- and stereoselectivity [33]. We attempted a rationalization of the photochemical behaviour on the basis of semiempirical calculations, showing that: (a) the observed high regioselectivity could be explained on the basis of a frontier orbitals control, (b) the interaction of the excited triplet state of the furylacrylate ester with another molecule of the reagent in the ground state gave the corresponding biradical intermediates where the more stable one has the same stereochemistry of the most abundant product, and (c) the following ring closure to obtain the products are under kinetic control [34,35]. In the meantime, several accurate approaches to the treatment of photochemical dimerization occurred. For example, the CASSCF/CASPT2 study of the photodimerization of cytosine showed the most probable evolution of the reaction along the potential energy hypersurfaces [36,37,38]. In this paper, we wish to report a DFT study of these reactions which not only explains the observed photochemical behavior, confirming the results obtained by using simple semiempirical methods, but also shows that a different approach has to be followed in order to explain the ring closure reaction. Density functional theory was largely used in the treatment of diradical species [39,40,41].

## 2. Computational Details

Gaussian09 has been used for the discussions about the computed geometries [42]. All the computations were based on the Density Functional Theory (DFT) [43] and Time-Dependent DFT (TD-DFT) [44,45] by using the B3LYP hybrid xc functional [46]. Geometry optimizations and TD-DFT results from the Gaussian09 program have been obtained at the B3LYP/6-31G+(d,p) level of approximation. Geometry optimization were performed in some cases at B3LYP/aug-cc-pVDZ level of approximation. Geometry optimizations were performed with default settings on geometry convergence (gradients and displacements), integration grid and electronic density (SCF) convergence. Redundant coordinates were used for the geometry optimization as produced by the Gaussian09 program. Analytical evaluation of the energy second derivative matrix w.r.t. Cartesian coordinates (Hessian matrix) at the B3LYP/6-31G+(d,p) and B3LYP/aug-cc-pVDZ levesl of approximation confirmed the nature of minima on the energy surface points associated to the optimized structures.

## 3. Results and Discussion

**Scheme 3:**
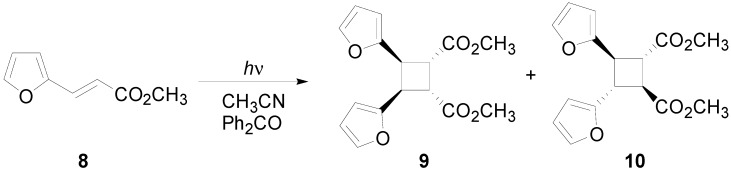
Photochemical dimerization of furylacrylate derivatives.

The irradiation of methyl 3-(2-furyl)acrylate (**8**) in acetonitrile in the presence of benzophenone as sensitizer furnished a mixture of two compounds **9** and **10** in 61% and 27%, respectively (Scheme 3) [31,32]. Kinetic and spectroscopic properties are in agreement with a mechanism where the furylacrylate dimerization occurs in the triplet state of the molecule and where this triplet state is obtained via energy transfer from benzophenone [47].

The reaction showed a high regioselectivity and a good stereoselectivity (only two stereoisomers were obtained when eleven ones were possible). To explain the observed regio- and stereoselectivity we performed DFT and TD-DFT calculations at B3LYP/6-31G+(d,p) and B3LYP/aug-cc-pVDZ level of theory using Gaussian09 [42].

**Figure 1:**
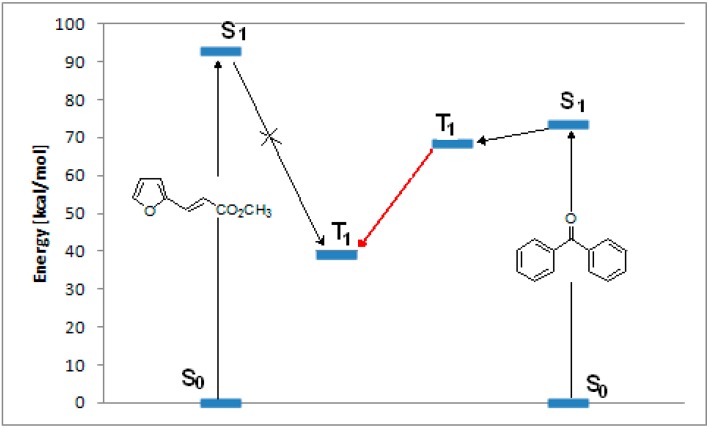
Energy transfer between benzophenone and **8**.

TD-DFT calculations on **8** showed that the S_1_ state had an energy of 93.30 kcal·mol^−1^, corresponding to a n→π* transition at 306 nm (experimental 299 nm) between the HOMO-1 to the LUMO (Figure 1). Experimental results showed that this excited singlet state was able to give only *trans*→*cis* isomerization [47]. On the basis of these experimental results, the triplet state, found at 39.33 kcal·mol^−1^, cannot be populated through an intersystem crossing from the excited singlet state (Figure 1). Benzophenone showed a triplet state with an energy of 68.6 kcal·mol^−1^ (Figure 1). It can act as a sensitizer of the excited triplet state of **8** (Figure 1).

**Figure 2:**
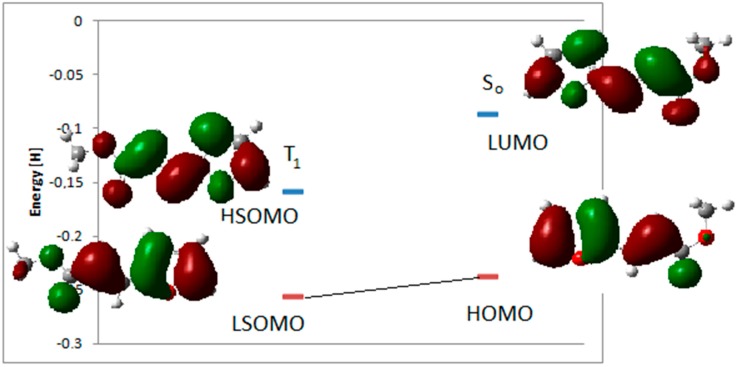
Interaction between frontier orbitals of triplet excited state of **8** and **8** in its ground state.

**Figure 3:**
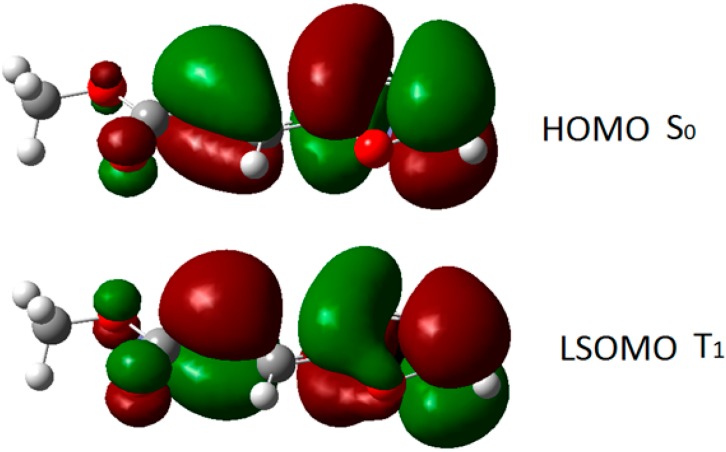
Frontier orbitals in the photodimerization of furylacrylate derivatives.

The triplet state of **8** can interact with the singlet state of another molecule of **8**. Admitting a frontier orbitals control of the reaction, the best interaction was observed between the LSOMO of the triplet **8** and the HOMO of the singlet state (Figure 2). Furthermore, we could see that there was a total superposition between the LSOMO of the triplet state and the HOMO of the singlet state (Figure 3, Table 1). The complete superposition between LSOMO and HOMO represents the best explanation of the observed regioselectivity.

**Figure 4:**
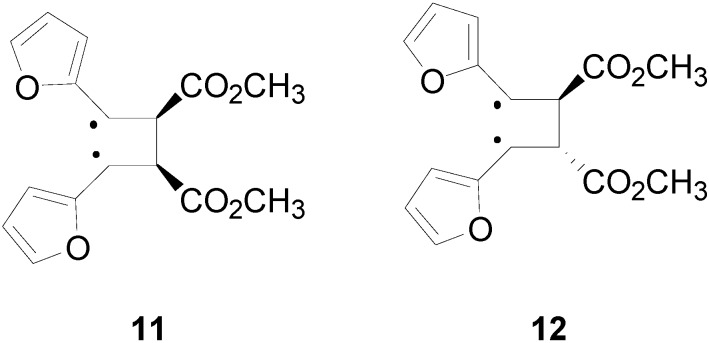
Biradical intermediates in the photochemical dimerization of **8**.

The superposition between two molecules of **8** allowed the formation of two possible biradical intermediates, where the relative configuration of two carbon atom on the future cyclobutane ring was defined. *Cis* or *trans* biradical intermediates **11** and **12** could be obtained (Figure 4).

**Table 1:** Atomic coefficients of the *p*_z_ orbitals in the HOMO of the S_0_ state and in the LSOMO of the T_1_ state of **8**. 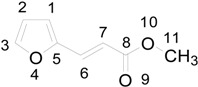

Atom	S_0_	T_1_
1	−0.22321	−0.21131
2	0.16272	0.11673
3	0.30938	0.31608
4	−0.02587	0.04881
5	−0.29943	−0.25936
6	0.12484	0.07397
7	0.29891	0.36801
8	0.02106	0.03188
9	−0.17997	−0.18654
10	−0.03305	−0.12168
11	−0.01552	0.04035

**Figure 5:**
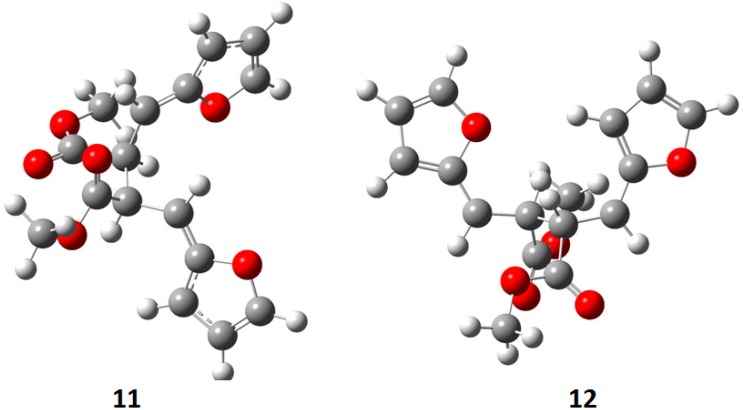
Actual structure of most stable conformer the biradical intermediates in the photochemical dimerization of **8**.

In Figure 5 the actual structure of the most stable conformer of the biradicals is showed. It is noteworthy that the *cis* biradical intermediate **11** showed that the bond between α carbon atom of the furan ring and the radical carbon atom was a double bond: it showed a large delocalization of the radical between the aromatic rings and the radical carbons atoms.

The *cis* biradical intermediate **11** was more stable than the *trans* **12** by 2.2 kcal·mol^−1^. This difference in the stability of the biradical intermediate could explain the observed ratio between the products. In fact, the main product of the reaction derived from the *cis* biradical while the minor product derived from the *trans* biradical.

**Figure 6:**
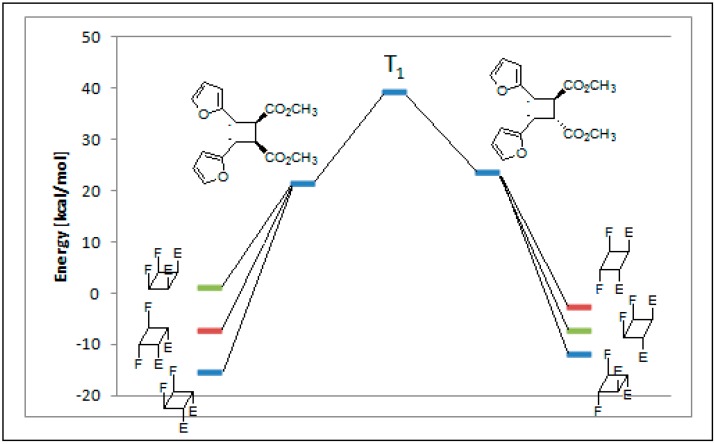
Relative energy of biradical intermediates **11** and **12** and of all the possible cyclobutane derivatives (DFT/B3LYP/6-31G+(d,p) level). F = 2-furyl; E = -CO_2_CH_3_.

The coupling between the radical carbon atoms after intersystem crossing allowed the formation of the cyclobutane ring. The energy of all the possible cyclobutanes that can be obtained starting from the biradical intermediates **11** and **12** was calculated. The results are collected in Figure 6 and Table 2.

We can see that, at 6-31G+(d,p) level, the observed products in the reactions were the more stable cyclobutane derivatives that can be obtained starting from the *cis* and *trans* biradical intermediates.

We performed also some calculations by using a different basis set (aug-cc-pVDZ). This basis set is larger than 6-31G+(d,p), allowing, in theory, to obtain more accurate results; however, it has to be noted some results appeared where aug-cc-pVDZ basis set was not able to give satisfactory results [48]. Anyway, we decided to compare the results obtained with this basis set, also considering that, in the calculation of the energy of the biradical intermediates **11** and **12**, aug-cc-pVDZ basis set gave results in agreement with those reported above (2.4 kcal·mol^−1^). In this case the most stable dimer was that corresponding to entry 2 of Table 2. It was a *trans-anti* dimer. It has to be noted that, in this case, the most stable *cis* dimer was not that at the entry 1 of the Table 2, but that of entry 5. On the basis of these results, considering that different basis sets gave different orders of stability, we were not able to verify if the assumption that the reaction gave the more stable ones can be accepted. On the basis of the above reported considerations on the problems encountered in the use of some basis sets, we cannot establish which method is able to give more accurate results. We can only note that they give different results. Furthermore, Table 2 showed also that the most important contribution to the dimers stability is an enthalpic one.

**Table 2:** Energy of suitable cyclobutane derivatives that can be obtained in the photochemical dimerization of **8**.

Entry	Cyclobutane	Relative Energy (kcal·mol^−1^)			H [kcal·mol^−1^]	G [kcal·mol^−1^]
		*a*	*b*	*c*	aug-cc-pVDZ	
1	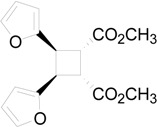	0	0.00	5.62	5.42	5.84
2	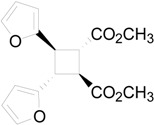	1010	3.73	0.00	0.00	0.00
3	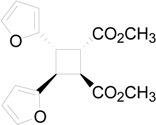	34	12.87	8.94	8.62	9.42
4	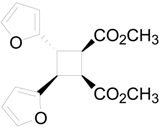	214	8.34	3.63	3.90	4.69
5	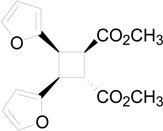	21	8.26	4.51	4.37	5.09
6	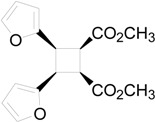	247	16.69	12.55	12.30	14.05

*a*: at AM1-UHF level (Ref. 21b); *b*: at DFT/B3LYP/6-31G+(d,p) level.; *c*: at DFT/B3LYP/aug-cc-pVDZ level.

In conclusion, our calculations showed that the regiochemistry of the reaction can be explained considering the superposition between the LSOMO of the triplet state of **8** and the HOMO of the same molecule in the ground state. The relative stability between the biradical intermediates accounts for the observed stereoselectivity while the use of different basis sets does not allow us to assert that the effective products obtained in the reaction depend on the relative stability of cyclobutane derivatives (see below). To confirm this behaviour we tested this methodology with another reaction. Some years ago we reported the photochemical dimerization of the ester of urocanic acid [33].

**Scheme 4:**
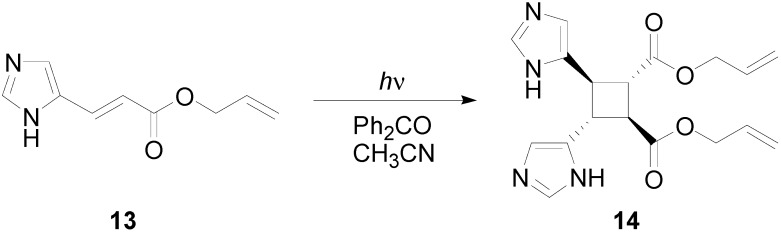
Photochemical dimerization of allyl urocanate

In particular, we reported that allyl urocanate **13** gave the corresponding dimer **14** with high regioselectivity and high stereoselectivity (Scheme 4).

The TD-B3LYP calculated first excited singlet state of **13** showed an energy of 95.30 kcal·mol^−1^, corresponding to a transition at 300 nm due to a HOMO→LUMO transition. The triplet state has been determined at 51.19 kcal·mol^−1^. Therefore, benzophenone could act as sensitizer also in this case populating the first excited allyl urocanate triplet state. The HOMO of **13** showed an energy of −0.23382 H while the LUMO was found at −0.07697 H. The triplet state of **13** showed a LSOMO at −0.25870 H while the HSOMO was found at −0.15706 H. Therefore, the best interaction between the frontier orbitals was found between the HOMO of the ground state and the LSOMO of the triplet state.

Table 3 collects the atomic coefficients of HOMO of **13** in the ground state and the LSOMO of the same compound in its triplet state. We can see that, with the exception of atom 4 and 8, we observe a good superposition between the two orbitals, in agreement with a head-to-head regiochemical control of the reaction.

**Table 3:** Atomic coefficients of the *p*_z_ orbitals in the HOMO of the S_0_ state and in the LSOMO of the T_1_ state of **13**. 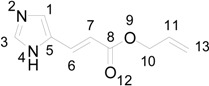

Atom	S_0_	T_1_
1	0.33944	0.15839
2	−0.06852	−0.08282
3	−0.25121	−0.17719
4	−0.15484	0.01599
5	0.28130	0.16271
6	−0.11141	−0.08653
7	−0.20234	−0.19414
8	−0.00484	0.00584
9	0.04775	0.11575
10	−0.01751	−0.08307
11	0.00554	0.13532
12	0.10280	0.08023
13	0.00708	0.14128

**Figure 7:**
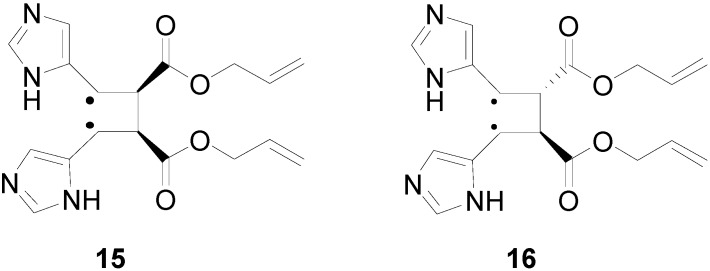
Biradical intermediates in the photochemical dimerization of **13**.

The superposition between two molecules of **13** allowed the formation of two possible biradical intermediates. We can obtain the *cis* and *trans* biradical intermediates **15** and **16** (Figure 7).

**Figure 8:**
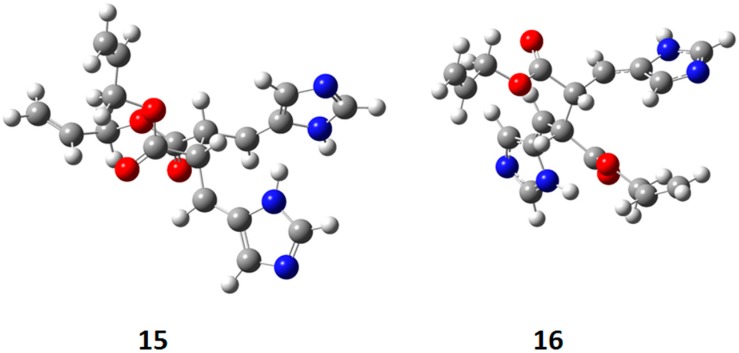
Actual structure of most stable conformer the biradical intermediates in the photochemical dimerization of **13**.

In Figure 8 the actual structure of the most stable conformers of **15** and **16** is shown. In this case, the observed delocalization of the radical on the aromatic ring, showed in **11**, was not present. On the contrary, this delocalization was present in the *trans* biradical **16**.

The *cis* biradical intermediate **15** was less stable than the *trans* one **16** for 29.99 kcal·mol^−1^. This difference in the stability of the biradical intermediate can explain the observed that only a *trans* cyclobutane derivate was obtained. Furthermore, the energy of *cis* biradical intermediate (66.02 kcal·mol^−1^) is higher than the energy of the triplet state (51.19 kcal·mol^−1^). Therefore, the *cis* biradical intermediate **15** cannot be obtained in the reaction mixture.

**Figure 9:**
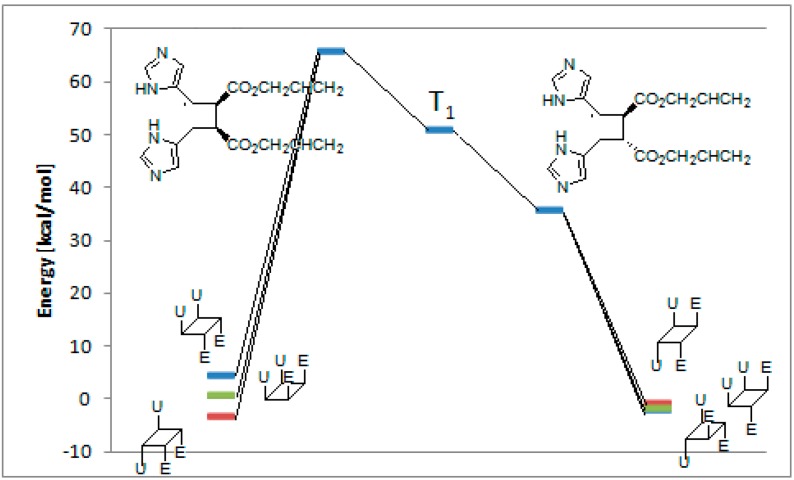
Relative energy of biradical intermediates **15** and **16** and of all the possible cyclobutane derivatives (DFT/B3LYP/6-31G+(d,p) level). U = 5-imidazolyl; E = -CO_2_CHCH=CH_2_.

The coupling between the radical carbon atoms after intersystem crossing allowed the formation of the cyclobutane ring. We calculated the energy of all the possible cyclobutanes that can be obtained starting from the biradical intermediates **15** and **16**. The results are collected in Figure 9 and Table 4.

We can see that the *cis* biradical intermediate cannot be formed and the product can be obtained only by the *trans* biradical intermediate. However, the energy of the cyclobutane derivatives that can be obtained starting from **16** are very close each other. 

In this case, we considered also the possible effect of the solvent (acetonitrile) on the relative stability of the dimers. The results are presented in Table 4. In this case, the most stable dimer was that at the entry 2 of the Table: however, the energy difference between the dimers did not account for the observed selectivity.

**Table 4:** Energy of suitable cyclobutane derivatives that can be obtained in the photochemical dimerization of **13**.

Entry	Cyclobutane	Relative Energy (kcal·mol^−1^)	
		6-31G+(d,p)	Solvent
1	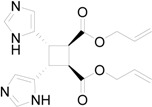	4.48	3.89
2	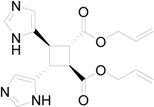	1.30	0.00
3	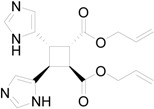	2.58	1.63
4	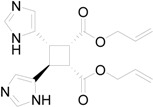	0.00	0.25
5	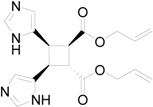	1.48	2.43
6	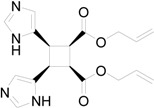	4.21	4.59

Therefore, on the basis of the unresolved questions appeared in the cyclization reaction in both furylacrylates and urocanates, a more accurate examination of the ring closure reaction has been performed.

**Figure 10:**
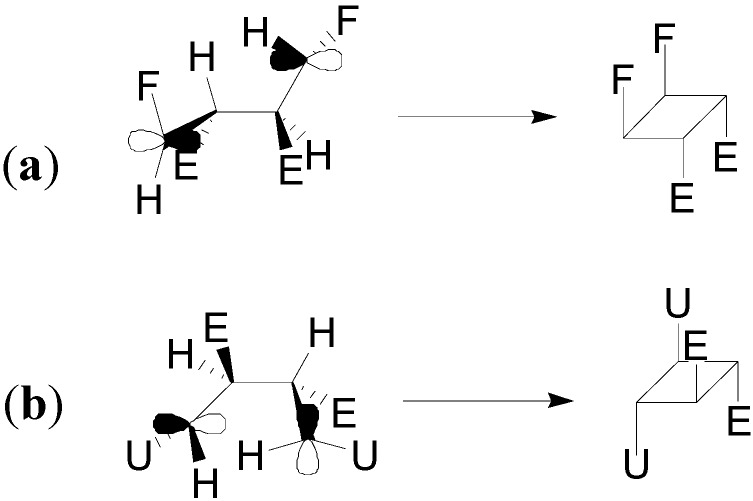
Coupling of LSOMO and HSOMO in the biradical intermediates **11** and **16**. (**a**) Ring closure in the biradical **11**; (**b**) Ring closure in the biradical **16**.

We examined the LSOMO and the HSOMO of the biradical intermediates **11** and **16**. Considering the atomic coefficients at the radical carbon atom in these orbitals, the coupling between these orbitals, after the intersystem crossing, can occur in the case of **11** (Figure 10A) giving only the product **9**, while in the case of **16** (Figure 10B) the coupling can give only the product **14**. Obviously, the biradical intermediate **12** will give the dimer **10**. This type of approach has been frequently used in order to justify the stereochemistry of [2+2] cycloaddition reactions [49,50,51].

**Figure 11:**
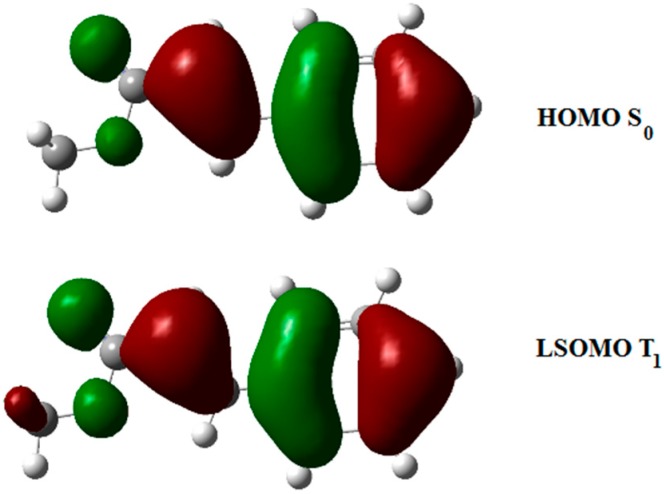
Frontier orbitals in the photodimerization of methyl cinnamate.

**Figure 12:**
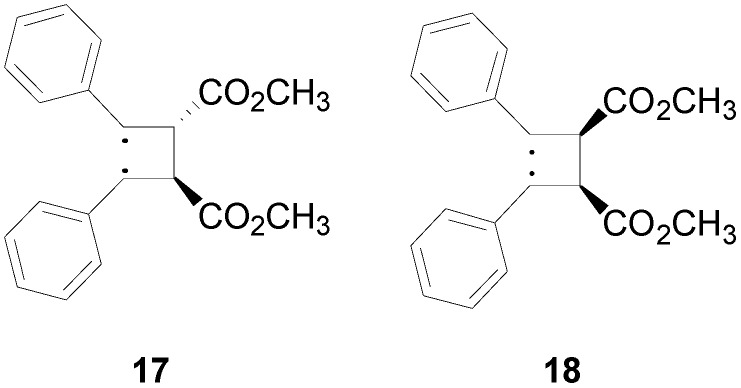
Biradical intermediates in the photochemical dimerization of methyl cinnamate.

Finally, we tested the above described approach in the photochemical dimerization of ethyl cinnamate. In this case, a mixture of **5** and **6** were obtained (Scheme 2) [14,15]. We performed our calculations on the methyl cinnamate. The reaction occurred in the triplet state and, also in this case, we can observe a complete superposition of the HOMO of methyl cinnamate with the LSOMO of triplet state of another molecule of the same compound (Figure 11). The interaction between these orbitals allowed the formation of only the head-to-head dimers. The coupling between these two molecules could afford the corresponding *cis* and *trans* biradical intermediates **17** and **18** (Figure 12).

The *trans* biradical intermediate **17** was more stable than the *cis* one **18** by 10.83 kcal·mol^−1^, in agreement with the observed products. Furthermore, considering the subsequent coupling of the biradicals, the atomic coefficents at the radical carbon atoms in the HSOMO and in the LSOMO of **17** allowed the formation of a cyclobutane where a *trans-anti-trans* relationship is present between the substituents. On the other hand, the atomic coefficents at the radical carbon atoms in the HSOMO and in the LSOMO of **18** allowed the formation of a cyclobutane where a *cis-anti-cis* relationship is present.

## 4. Conclusions

In this paper we have shown that regio- and stereochemical behaviour of heteroarylacrylates dimerization in solution can be understood by using simple calculation procedures. The reaction is a sensitized reaction from the excited triplet state and the regiochemistry is controlled by the frontier orbitals superposition of the two reagents. The coupling allows the formation of the most stable biradical intermediate, thus inducing a control in the possible stereochemical behavior. Furthermore, the coupling of the two radical carbon atoms explained the observed stereochemistry.
